# Supplementary material for: State-by-state influenza outbreaks and oversee: A Markov chain study of California and North Carolina, USA
Source: PLOS Glob Public Health. 2025 Sep 18;5(9):e0005135. doi: 10.1371/journal.pgph.0005135 (PMC12445519; doi:10.1371/journal.pgph.0005135)
Supplement: S1 Appendix — (PDF) [file pgph.0005135.s001.pdf]

Supporting information

S1 Appendix. Demonstrations of the basic reproduction number and fixed points. This section presents simulations of the basic reproduction number and the fixed points, which include the disease-free equilibrium and the endemic equilibrium.

Recall the mathematical model and we have,

$$\mathbb{Y}' = F(Y,t),\ \mathbb{Y}(0) = \mathbb{Y}_0. \tag{1}$$

where, the initial conditions, $\mathbb{Y}_0 = (S_0, E_0, I_0, R_0)$, and,

$$F(Y,t) = [\mu N - \frac{\beta IS}{N} + \omega R - \mu S, \frac{\beta IS}{N} - \sigma E - \mu E, \sigma E - \gamma I - \mu I, \gamma I - \omega R - \mu R].$$

Basic Reproduction Number. The basic reproduction number ($\mathscr{R}_0$), is defined as the average number of secondary cases of an infectious disease arising from a typical case in a susceptible population [1]. When $\mathscr{R}_0 < 1$, the system is asymptotically stable and the disease-free equilibrium point exists. The endemic equilibrium $\mathscr{R}_0 > 1$, and the disease may spread out through the population [2, 3]. When modeling infectious diseases, a critical threshold analysis is the fundamental reproduction number. The ability of an infectious disease to spread quickly through a community can be usefully assessed [4, 5]. Basic reproduction numbers can be computed using a number of methods. Here, it is computed using the next-generation matrix technique.

Calculation of $\mathscr{R}_0$. $\mathscr{R}_0$ is the spectral radius of the matrix FV^{-1}. Let $f = \dfrac{\beta SI}{N}$, and $g = 0$. Then,

$$f^* = \begin{bmatrix} \frac{\beta SI}{N} \\ 0 \end{bmatrix},\ f_1 = -(\sigma+\mu)E,\ f_2 = \sigma E - (\gamma+\mu)I$$

such that

$$v = \begin{bmatrix} -(\sigma+\mu)E \\ \sigma E - (\gamma+\mu)I \end{bmatrix},\ F = \begin{bmatrix} \frac{\partial f}{\partial E} & \frac{\partial f}{\partial I} \\ \frac{\partial g}{\partial E} & \frac{\partial g}{\partial I} \end{bmatrix} = \begin{bmatrix} 0 & \frac{\beta S}{N} \\ 0 & 0 \end{bmatrix},$$

and

$$V = \begin{bmatrix} \frac{\partial f_1}{\partial E} & \frac{\partial f_1}{\partial I} \\ \frac{\partial g_1}{\partial E} & \frac{\partial g_1}{\partial I} \end{bmatrix} = \begin{bmatrix} (\sigma+\mu) & 0 \\ -\sigma & (\gamma+\mu) \end{bmatrix}.$$

Therefore,

$$V^{-1} = \begin{bmatrix} (\sigma+\mu)^{-1} & 0 \\ \sigma(\sigma+\mu)^{-1}(\gamma+\mu)^{-1} & (\gamma+\mu)^{-1} \end{bmatrix}.$$

Hence,

$$FV^{-1} = \begin{bmatrix} 0 & \frac{\beta S}{N} \\ 0 & 0 \end{bmatrix} \begin{bmatrix} \frac{1}{\sigma+\mu} & 0 \\ \frac{1}{\sigma(\sigma+\mu)(\gamma+\mu)} & \frac{1}{\gamma+\mu} \end{bmatrix} = \begin{pmatrix} \frac{\beta S\sigma}{N(\sigma+\mu)(\gamma+\mu)} & \frac{\beta S}{N(\gamma+\mu)} \\ 0 & 0 \end{pmatrix}.$$

The spectral radius of FV^{-1} is,

$$\rho(FV^{-1}) = \frac{\sigma\beta S}{N(\sigma+\mu)(\gamma+\mu)}.$$

The susceptible number of the population can be at most N, i.e $S = N$, which yields,

$$\mathscr{R}_0 = \frac{\sigma\beta}{(\sigma+\mu)(\gamma+\mu)}.$$

Determination of Fixed Points. The equilibrium point can be calculated by setting all the 1st derivative terms to zero of the equation (1). Thus,

$$\begin{cases} \mu N - \dfrac{\beta IS}{N} + \omega R - \mu S = 0. \\ \dfrac{\beta IS}{N} - \sigma E - \mu E = 0. \\ \sigma E - \gamma I - \mu I = 0. \\ \gamma I - \omega R - \mu R = 0. \end{cases} \tag{2}$$

Disease-Free Equilibrium (DFE). Disease-free equilibrium can be obtained by assuming $I = 0$. Since there will be no disease at the disease-free equilibrium point. Hence, the equation (2) becomes,

$$\begin{cases} \mu N + \omega R_0 - \mu S_0 = 0. \\ -(\sigma+\mu)E_0 = 0. \\ \sigma E_0 = 0. \\ -(\omega+\mu)R_0 = 0. \end{cases} \tag{3}$$

Solving this system of linear equations, we get $S_0 = N,\ E_0 = 0,\ I_0 = 0,\ R_0 = 0$. Hence, the equilibrium point is $P_0 = (N, 0, 0, 0)$.

Thus, the basic reproduction number at P_0 is $\dfrac{\sigma\beta}{(\sigma+\mu)(\gamma+\mu)}$.

Endemic Equilibrium. At the endemic equilibrium point, at least one of the infected groups is non-zero i.e, $I \neq 0$. Let $P_1 = (S_1, E_1, I_1, R_1)$ be any arbitrary endemic equilibrium point. Then equation (2) becomes,

$$\begin{cases} \mu N - \dfrac{\beta I_1 S_1}{N} + \omega R_1 - \mu S_1 = 0. \\ \dfrac{\beta I_1 S_1}{N} - (\sigma+\mu)E_1 = 0. \\ \sigma E_1 - (\gamma+\mu)I_1 = 0. \\ \gamma I_1 - (\omega+\mu)R_1 = 0. \end{cases} \tag{4}$$

which can be written as,

$$\omega R_1 - \frac{\beta I_1 S_1}{N} = \mu S_1 - \mu N. \tag{5}$$

$$\frac{\beta I_1 S_1}{N} = (\sigma+\mu)E_1. \tag{6}$$

$$\sigma E_1 = (\gamma+\mu)I_1. \tag{7}$$

$$\gamma I_1 = (\omega+\mu)R_1. \tag{8}$$

From (8), we can write,

$$I_1 = \frac{(\omega+\mu)}{\gamma}R_1. \tag{9}$$

From (7),

$$E_1 = \frac{(\gamma+\mu)}{\sigma} I_1 = \frac{(\gamma+\mu)}{\sigma}\frac{(\omega+\mu)}{\gamma} R_1. \tag{10}$$

From (6),

$$\frac{\beta I_1 S_1}{N} = (\sigma+\mu)E_1 = (\sigma+\mu)\frac{(\gamma+\mu)}{\sigma} I_1$$

$$\Rightarrow S_1 = \frac{N(\sigma+\mu)(\gamma+\mu)}{\sigma\beta} = \frac{N}{\mathscr{R}_0}. \tag{11}$$

where, $\mathscr{R}_0 = \dfrac{\sigma\beta}{(\sigma+\mu)(\gamma+\mu)}$, and (5) can be written as,

$$\omega R_1 - \frac{\beta}{N}\frac{(\omega+\mu)}{\gamma} R_1 \frac{N}{\mathscr{R}_0} = \mu\frac{N}{\mathscr{R}_0} - \mu N$$

$$\Rightarrow R_1 = \frac{(\mathscr{R}_0 - 1)\,\mu N}{\left[\dfrac{\beta(\omega+\mu)}{\gamma} - \omega\mathscr{R}_0\right]}. \tag{12}$$

Thus,

$$S_1 = \frac{N}{\mathscr{R}_0},$$

$$E_1 = \left(\frac{(\gamma+\mu)(\omega+\mu)}{\sigma\gamma}\right)\left(\frac{(\mathscr{R}_0-1)\,\mu N}{\left[\dfrac{\beta(\omega+\mu)}{\gamma} - \omega\mathscr{R}_0\right]}\right) = \left(\frac{(\mathscr{R}_0-1)\,\mu N(\gamma+\mu)(\omega+\mu)}{\sigma\left[\beta(\omega+\mu) - \omega\gamma\mathscr{R}_0\right]}\right),$$

$$I_1 = \left(\frac{\omega+\mu}{\gamma}\right)\left(\frac{(\mathscr{R}_0-1)\,\mu N}{\left[\dfrac{\beta(\omega+\mu)}{\gamma} - \omega\mathscr{R}_0\right]}\right) = \left(\frac{(\mathscr{R}_0-1)\,\mu N(\omega+\mu)}{\left[\beta(\omega+\mu) - \omega\gamma\mathscr{R}_0\right]}\right),$$

$$R_1 = \left(\frac{(\mathscr{R}_0-1)\,\mu N}{\left[\dfrac{\beta(\omega+\mu)}{\gamma} - \omega\mathscr{R}_0\right]}\right) = \frac{(\mathscr{R}_0-1)\,\gamma\mu N}{\left[\beta(\omega+\mu) - \omega\gamma\mathscr{R}_0\right]}.$$

Thus, the endemic equilibrium point is,

$$P_1 = \left(\frac{N}{\mathscr{R}_0}, \frac{(\mathscr{R}_0-1)\,\mu N(\gamma+\mu)(\omega+\mu)}{\sigma\left[\beta(\omega+\mu) - \omega\gamma\mathscr{R}_0\right]}, \frac{(\mathscr{R}_0-1)\,\mu N(\omega+\mu)}{\left[\beta(\omega+\mu) - \omega\gamma\mathscr{R}_0\right]}, \frac{(\mathscr{R}_0-1)\,\gamma\mu N}{\left[\beta(\omega+\mu) - \omega\gamma\mathscr{R}_0\right]}\right).$$

References

1. Guerra FM, Bolotin S, Lim G, Heffernan J, Deeks SL, Li Y, Crowcroft NS. The basic reproduction number (R0) of measles: a systematic review. Lancet Infect Dis. 2017;17(12):e420-e428.

2. Martcheva M. An introduction to mathematical epidemiology. New York: Springer; 2015. p. 9-31. (Vol. 61).

3. Akhi AA, Tasnim F, Akter S, Kamrujjaman M. A mathematical model of a diphtheria outbreak in Rohingya settlement in Bangladesh. J Mahani Math Res Cent. 2023;12(2):547-63. doi:10.22103/jmmr.2023.19459.1256.

4. Nishiura H. Correcting the actual reproduction number: a simple method to estimate R0 from early epidemic growth data. Int J Environ Res Public Health. 2010;7(1):291-302.

5. Kamrujjaman M, Mahmud MS, Ahmed S, Qayum MO, Alam MM, Hassan MN, Bulut U. SARS-CoV-2 and Rohingya refugee camp, Bangladesh: uncertainty and how the government took over the situation. Biology. 2021;10(2):124.
