# Supplementary material for: State-by-state influenza outbreaks and oversee: A Markov chain study of California and North Carolina, USA
Source: PLOS Glob Public Health. 2025 Sep 18;5(9):e0005135. doi: 10.1371/journal.pgph.0005135 (PMC12445519; doi:10.1371/journal.pgph.0005135)
Supplement: S2 Appendix — (PDF) [file pgph.0005135.s002.pdf]

Supporting information

S2 Appendix. Analytical stability and sensitivity study: This section provides a brief discussion on the stability of the disease-free and endemic equilibria, along with a sensitivity analysis of the proposed model.
Based on the mathematical model, we have,

$$\mathbb{Y}' = F(Y,t),\ \mathbb{Y}(0) = \mathbb{Y}_0. \tag{1}$$

where, the initial conditions, $\mathbb{Y}_0 = (S_0, E_0, I_0, R_0)$, and,

$$F(Y,t) = [\mu N - \frac{\beta IS}{N} + \omega R - \mu S, \frac{\beta IS}{N} - \sigma E - \mu E, \sigma E - \gamma I - \mu I, \gamma I - \omega R - \mu R].$$

Stability Analysis. The local asymptotic stability (LAS) of the endemic equilibrium (EE) and disease-free equilibrium (DFE) points are examined in this section.

Theorem 1. *If the basic reproduction number $\mathscr{R}_0 < 1$, the system's disease-free equilibrium point is stable and unstable when the basic reproduction number $\mathscr{R}_0 > 1$.*

Proof. At the disease-free equilibrium point, the Jacobian matrix of the system (1) is,

$$J(P_0) = \begin{pmatrix} -\mu & 0 & -\beta & \omega \\ 0 & -(\sigma+\mu) & \beta & 0 \\ 0 & \sigma & -(\gamma+\mu) & 0 \\ 0 & 0 & \gamma & -(\omega+\mu) \end{pmatrix}.$$

The characteristic equation of the Jacobian matrix is $|J(P_0) - \lambda I| = 0$, where I is the identity matrix. Thus, the determinant of the characteristic matrix is,

$$\text{Det}(J(P_0) - \lambda I) = \begin{pmatrix} -(\mu+\lambda) & 0 & -\beta & \omega \\ 0 & -(\sigma+\mu+\lambda) & \beta & 0 \\ 0 & \sigma & -(\gamma+\mu+\lambda) & 0 \\ 0 & 0 & \gamma & -(\omega+\mu+\lambda) \end{pmatrix}.$$

Expanding concerning the first column and then the third column, we will get the following characteristic equation:

$$(\mu+\lambda)(\omega+\mu+\lambda)[(\lambda+\gamma+\mu)(\lambda+\sigma+\mu) - \sigma\beta] = 0. \tag{2}$$

Two eigenvalues of equation (2) are $\lambda_1 = -\mu$ and $\lambda_2 = -(\omega+\mu)$ which are less than zero. The other two eigenvalues satisfy the equation,

$$\lambda^2 + A\lambda + B = 0. \tag{3}$$

Where $A = \gamma + \sigma + 2\mu$, and $B = (\gamma+\mu)(\sigma+\mu) - \sigma\beta$.

Since σ, γ, μ are positive, A is always positive. B will be positive if and only if $\frac{\sigma\beta}{(\gamma+\mu)(\sigma+\mu)} < 1$ i.e, $\mathscr{R}_0 < 1$.

Hence, the disease-free equilibrium point P_0 is locally asymptotically stable if $\mathscr{R}_0 < 1$ and unstable if $\mathscr{R}_0 > 1$ according to Hurwitz–Routh criterion [1]. □

Theorem 2. *The endemic equilibrium point of the system is stable if the basic reproduction number $\mathscr{R}_0 > 1$.*

Proof. The Jacobian matrix of the system (1) at endemic equilibrium point $P_1 = (S_1, E_1, I_1, R_1)$ is,

$$\mathbb{J}(P_1) = \begin{pmatrix} -\mu - \dfrac{\beta I_1}{N} & 0 & \dfrac{-\beta S_1}{N} & \omega \\ \dfrac{\beta I_1}{N} & -(\sigma+\mu) & \dfrac{\beta S_1}{N} & 0 \\ 0 & \sigma & -(\gamma+\mu) & 0 \\ 0 & 0 & \gamma & -(\omega+\mu) \end{pmatrix}.$$

The determinant of the matrix is,

$$\text{Det}(\mathbb{J}) = \frac{\mu(\mu+\omega)\left[-\beta^2\sigma(\mu+\omega) + \mu\mathscr{R}_0^2(\mu+\sigma)\left\{\beta(\mu+\omega) - \gamma\omega\right\} + \mathbb{M} + \beta\gamma\mathscr{R}_0\sigma\omega\right]}{\mathscr{R}_0\left[\beta(\mu+\omega) - \gamma\mathscr{R}_0\omega\right]} > 0$$

where,

$$\mathbb{M} = \gamma\mathscr{R}_0\left\{\beta\sigma\omega + \beta\mu\mathscr{R}_0(\mu+\sigma+\omega) - \gamma\mathscr{R}_0\omega(\mu+\sigma)\right\}$$

The characteristic equation of the Jacobian matrix is $|\mathbb{J}(P_1) - \lambda I| = 0$, where I is the identity matrix.

This characteristic equation satisfies the following quadratic equation,

$$\lambda^4 + A\lambda^3 + B\lambda^2 + C\lambda + D = 0. \tag{4}$$

Where

$A = -(a_1 + d_4 + b_2 + c_3)$.

$B = a_1 d_4 + b_2 c_3 - \sigma c_2 + a_1 b_2 + d_4 b_2 + c_3 a_1 + d_4 c_3$.

$C = -[a_1 d_4(b_2 + c_3) + (b_2 c_3 - \sigma c_2)(a_1 + d_4) + \sigma a_2 c_1]$.

$D = a_1 d_4(b_2 c_3 - \sigma c_2) + \sigma a_2(c_1 d_4 - \omega\gamma)$.

and

$a_1 = -\left(\dfrac{\beta I_1}{N} + \mu\right)$, $c_1 = -\dfrac{\beta S_1}{N}$, $a_2 = \dfrac{\beta I_1}{N}$, $b_2 = -(\sigma+\mu)$, $c_2 = \dfrac{\beta S_1}{N}$, $c_3 = -(\gamma+\mu)$, $d_4 = -(\omega+\mu)$.

As a consequence of the Routh-Hurwitz criteria, every root of this bi-quadratic equation possesses a negative real part if and only if $A,\ B,\ C,\ D > 0$, $AB > C$ and $ABC > C^2 + A^2 D$ [2].

After substituting these values in A, B, C, and D and simplifying, we obtain,

$A = \dfrac{\beta I_1}{N} + 4\mu + \sigma + \omega + \gamma$.

$B = (\dfrac{\beta I_1}{N} + \mu)(3\mu + \sigma + \omega + \gamma) + (\gamma + 2\mu + \sigma)(\omega + \mu)$.

$C = \left(\dfrac{\beta I_1}{N} + \mu\right)(\gamma + 2\mu + \sigma)(\omega + \mu) + \left(\dfrac{\beta I_1}{N}\right)\left(\dfrac{\sigma\beta S_1}{N}\right)$.

$D = \dfrac{\sigma\beta I_1}{N}\left[\dfrac{\beta}{\mathscr{R}_0}(\omega+\mu) - \omega\gamma\right] = \dfrac{\sigma\beta I_1}{N\mathscr{R}_0}\left[\beta(\omega+\mu) - \omega\gamma\mathscr{R}_0\right] = \dfrac{\sigma\beta I_1}{N\mathscr{R}_0}\dfrac{(\mathscr{R}_0 - 1)\,\gamma\mu N}{R_1}$.

Clearly, A, B, C, and $D > 0$ if and only if $\mathscr{R}_0 > 1$.

Hence, by Descartes's rule of signs [3], equation (4) will have no positive real roots if $\mathscr{R}_0 > 1$.

For $\mathscr{R}_0 > 1$, all roots of the equation (4) will have negative real portions, meeting the requirements of the Routh–Hurwitz criterion [4]. Thus, the EE point will be locally asymptotically stable if $\mathscr{R}_0 > 1$. □

Discussion of Sensitivity Analysis. We present a concise discussion of each parameter case, focusing on the Partial Rank Correlation Coefficient (PRCC) values and their corresponding p-values from the Monte Carlo simulation.

The SEIRS model for Influenza analyzes the sensitivity of key parameters—contact rate (β), latency rate (σ), recovery rate (γ), loss of immunity rate (ω), and the birth/death rate (μ)—using Monte Carlo simulation, with probability distributions capturing their expected variability. The contact rate (β), which represents how frequently susceptible individuals come into contact with infectious individuals, is modeled using a uniform distribution between 0.1 and 0.5, reflecting its wide variability depending on population density and social behavior.

The latency rate (σ), which measures the rate at which exposed individuals become infectious, is assigned a normal distribution with a mean of 0.2 and a standard deviation of 0.05, as it typically varies around a central value determined by biological factors. Similarly, the recovery rate (γ), which describes how quickly infected individuals recover, is also modeled with a normal distribution, having a mean of 0.1 and a standard deviation of 0.02, as recovery rates are generally clustered around a central value but influenced by factors like treatment and healthcare access.

For the loss of immunity rate (ω), which reflects how quickly recovered individuals lose immunity and become susceptible again, we use a uniform distribution between 0.01 and 0.1 due to the variability in immunity loss across different populations. Finally, the birth/death rate (μ), which accounts for the natural demographic changes in the population, is given a normal distribution with a mean of 0.01 and a standard deviation of 0.002, as these rates tend to be stable but with some minor fluctuations. By assigning these distributions, the Monte Carlo simulation can generate random parameter values in each iteration, allowing for a thorough sensitivity analysis of the model's outcomes.

Many deterministic or stochastic mathematical models with continuous or discrete features are subjected to the LHS/PRCC process. We may ascertain the degree of uncertainty that an LHS parameter contributes to the prototype by using the PRCC and proportionate p-values that the partial rank correlation analysis gives us. The statistical significance and magnitude of the parameter's PRCC value suggest that imprecision is the parameter's contribution to model forecasting. The most significant parameters include tiny p-values (< 0.05) and large PRCC values (> 0.5 or < -0.5). The closer the PRCC value is at $+1$ or -1, the more the LHS parameter influences the outcome measure. The sign denotes how qualitatively different the input and output variables are. The LHS parameter is inversely related to the result measure if it has a negative sign.

The death rate, μ, shows a very strong negative PRCC with the susceptible compartment (PRCC = -0.9875, $p < 0.001$), indicating that changes in mortality have a major impact on reducing the size of the susceptible population. This parameter also has a significant negative effect on the recovered (R) compartment (PRCC = -0.8717, $p < 0.001$), demonstrating that mortality influences the size of the recovered group. Additionally, μ negatively affects the exposed (E) and infected (I) compartments with PRCC values of -0.5689 and -0.3123, respectively, showing it has a widespread impact across all compartments.

The recovery rate (γ) exhibits strong negative sensitivity in the exposed (E) and infected (I) compartments, with PRCC values of -0.6393 and -0.4859, respectively (both with highly significant p-values). This suggests that increasing the recovery rate significantly decreases the number of individuals in these states, as it accelerates the transition of exposed and infected individuals towards recovery. The negative PRCC for the recovered compartment (PRCC = -0.3163) implies a complex interaction, potentially indicating that higher recovery also slightly impacts the accumulation of recovered individuals.

The transmission rate (β) has a positive PRCC with the exposed (E) and infected (I) compartments (PRCC = 0.2882 and 0.2818, respectively), with very small p-values, showing that an increase in transmission directly increases the size of these populations, as expected in disease dynamics. The positive PRCC for the recovered compartment (PRCC = 0.3274) indicates that higher transmission eventually leads to more individuals moving into the recovered state after passing through the infected stage.

The progression rate (σ) shows moderate sensitivity in the exposed compartment (PRCC = -0.0678, $p < 0.05$), with a small negative effect, indicating that it slightly decreases the exposed population. Its impact on other compartments is minimal, suggesting that changes in σ do not significantly alter the system's dynamics outside of the exposed compartment.

The rate of loss of immunity (ω) exhibits very low sensitivity across all compartments, with PRCC values near zero and higher p-values, indicating that this parameter has a negligible influence on the model. This suggests that immunity loss plays a minimal role in affecting the dynamics of the susceptible, exposed, infected, and recovered compartments.

References

1. Kim D, Puig A, Rabiei F, Hawkins EJ, Hernandez TF, Sung CK. Optimization of SOX2 expression for enhanced glioblastoma stem cell virotherapy. Symmetry. 2024;16(9):1186.

2. Mohammad KM, Akhi AA, Kamrujjaman M. Bifurcation analysis of an influenza A (H1N1) model with treatment and vaccination. PLoS One. 2025;20(1):e0315280.

3. Anderson B, Jackson J, Sitharam M. Descartes' rule of signs revisited. Am Math Mon. 1998;105(5):447-51.

4. Kamrujjaman M, Saha P, Islam MS, Ghosh U. Dynamics of SEIR model: A case study of COVID-19 in Italy. Results Control Optim. 2022;7:100119.
