# Supplementary material for: State-by-state influenza outbreaks and oversee: A Markov chain study of California and North Carolina, USA
Source: PLOS Glob Public Health. 2025 Sep 18;5(9):e0005135. doi: 10.1371/journal.pgph.0005135 (PMC12445519; doi:10.1371/journal.pgph.0005135)
Supplement: Attachment — Submitted filename: Response_R1_PGPH-D-25-00597.pdf [file pgph.0005135.s005.pdf]

Md. Kamrujjaman, Ph.D.
Professor
Department of Mathematics
University of Dhaka
Dhaka-1000, Bangladesh
E-mail: kamrujjaman@gmail.com
kamrujjaman@du.ac.bd

May 29, 2025

Editor-in-Chief/Academic Editor
PLOS Global Public Health

Dear Professor

Please find the revised version of the manuscript (Manuscript ID: PGPH-D-25-00597) entitled: **"State-by-State Influenza Outbreaks and Oversee: A Markov Chain Study of California and North Carolina, USA"** and consider it for publication in the journal **PLOS Global Public Health**.

We have revised the manuscript according to the Editors/Reviewers comments and suggestions.

Authors' Note: The major corrections and new addendum are highlighted in red color texts in the revised manuscript.

Reviewer-1

Reviewer Comments and Corresponding Answers:
In this study, the authors present a robust SEIRS mathematical model to analyze influenza transmission dynamics, using data from influenza cases in California and North Carolina between October 1, 2023, and September 24, 2024. This comprehensive study combines both deterministic and stochastic frameworks, making it relevant for public health planning.

A few minor comments are provided below:

1. For clarity, it would be beneficial if the authors explicitly define all parameters and introduce a schematic of the transmission dynamics in Section 2 of the mathematical models. Alternatively, they could refer to Table 1 and Figure 2 in the same section.
Authors' response: Thanks for your advice. We have incorporated your suggestion. Please see Section 2, Pages 6 and 7, for the explicit parameter definitions and the

schematic of the transmission dynamics.

2. Several additional factors can affect transmission, including individuals' age, spatial variations of infectious agents, co-infections, and mobility. Does this model also take these into account? Please discuss the limitations of the model in detail.
   Authors' response: Thank you for bringing this up. While the current model does not incorporate age, spatial variations, co-infections, or mobility, we acknowledge that these factors can influence transmission dynamics significantly. Our focus in this study was on the essential components of disease spread, and these additional variables represent potential areas for future work. Detailed limitations and potential avenues for future research are presented in Section 7, Page 31-32. These changes are highlighted in red color in revised manuscript.
   "...In the current model, we assume a homogeneous population and do not account for factors such as age, demographic and spatial variations, mobility, migration, vaccination, quarantine measures, or environmental conditions. Future work could address these limitations by incorporating heterogeneous populations, mobility patterns, and more complex interactions, thereby enhancing the model's realism and predictive accuracy."

Reviewer-2

Reviewer Comments and Corresponding Answers:
Authors have explained the transmission of influenza with the SEIR mathematical model perfectly. The equations, plots and their findings establish very clearly the spread of influenza and they validate it with actual case studies of North Carolina and California. The article provides full clarity on each of their parameters, equations and models to explain the transmission dynamics of Influenza.

Minor comment:

1. I would be interested in seeing a summary figure like what we usually see in epidemiology papers giving counts of individuals say 100 individuals for example who are susceptible (S), then this 100 becomes what in each step of SEIR flow, i.e. at E, I and R. Also, if they can incorporate how basic reproduction number changes at each step of S, E, I and R and finally it becomes lowest as recovery rate (gamma) increases into this figure, that would be great if possible. This figure should be able to explain the big picture of the whole paper like a graphical abstract which can go into Conclusions maybe.
   Authors' response: Thank you for your valuable suggestion. Based on your advice, we have shown the relationship between the effective reproduction number and the population distribution across compartments (Section 4.2.2, Pages 15, 16), revealing a strong correlation. When the susceptible population is high and the recovered population low, the effective reproduction number exceeds 1 (around 3) due to higher transmission and lower recovery rates. Over time, as recovery increases and suscep-

tibility decreases, the effective reproduction number falls below 1, indicating disease control. To clarify, we have summarized all numerical results as follows (Pages 30, 31):

"We have plotted each compartment by varying different parameters such as recovery rate and transmission rate. These numbers enable us to see how slight variations in the rate of transmission or recovery impact the overall number of susceptible, exposed, infected, and recovered individuals. We found that while the susceptible, infected, and exposed population grows as the transmission rate increases, the recovered population increases as the recovery rate increases. Additionally, we have demonstrated the relationship between the basic reproduction number and changes in transmission and recovery rates. We have discovered that the basic reproduction number rises linearly with an increase in transmission rate and falls with an increase in recovery rate. Phase plane analysis helps us to visualize how populations interact dynamically. These spiral-shaped curves suggest a potential for recurrent outbreaks, but in the long run, the disease will likely remain under control. The boxplot analysis provides us with the range of the basic reproduction number that determines the maximum or minimum number of exposed population that an infected individual infects at the initial stage of an outbreak. We may infer from the examination of contour plots and heat maps that a lower transmission rate and a quicker recovery rate are important components in reducing the impact of the epidemic. To forecast the long-term dynamics of influenza infections in North Carolina and California, we have estimated parameters using the least squares method. To determine the most sensitive parameter, a sensitivity analysis has been carried out using the Monte Carlo technique. We can compare the stochastic and deterministic situations with the aid of the CTMC approach."
All these revisions are highlighted in red color in revised manuscript.

General: We thank all reviewers for their constructive suggestions and comments to improve the quality of the paper in the Acknowledgment section.

Yours sincerely,

Md. Kamrujjaman

Editor-in-Chief/Academic Editor
PLOS Global Public Health
